# A MAP6-Related Protein Is Present in Protozoa and Is Involved in Flagellum Motility

**DOI:** 10.1371/journal.pone.0031344

**Published:** 2012-02-15

**Authors:** Denis Dacheux, Nicolas Landrein, Magali Thonnus, Guillaume Gilbert, Annelise Sahin, Harald Wodrich, Derrick R. Robinson, Mélanie Bonhivers

**Affiliations:** 1 Microbiologie Fondamentale et Pathogénicité, Université de Bordeaux, UMR 5234, Bordeaux, France; 2 Microbiologie Fondamentale et Pathogénicité, CNRS, UMR 5234, Bordeaux, France; 3 Microbiologie Fondamentale et Pathogénicité, Institut Polytechnique de Bordeaux, UMR 5234, Bordeaux, France; University of Texas-Houston Medical School, United States of America

## Abstract

In vertebrates the microtubule-associated proteins MAP6 and MAP6d1 stabilize cold-resistant microtubules. Cilia and flagella have cold-stable microtubules but MAP6 proteins have not been identified in these organelles. Here, we describe *Tb*SAXO as the first MAP6-related protein to be identified in a protozoan, *Trypanosoma brucei*. Using a heterologous expression system, we show that *Tb*SAXO is a microtubule stabilizing protein. Furthermore we identify the domains of the protein responsible for microtubule binding and stabilizing and show that they share homologies with the microtubule-stabilizing Mn domains of the MAP6 proteins. We demonstrate, in the flagellated parasite, that *Tb*SAXO is an axonemal protein that plays a role in flagellum motility. Lastly we provide evidence that *Tb*SAXO belongs to a group of MAP6-related proteins (SAXO proteins) present only in ciliated or flagellated organisms ranging from protozoa to mammals. We discuss the potential roles of the SAXO proteins in cilia and flagella function.

## Introduction

The cytoskeleton of eukaryotic cells is essential to maintain cell structure and polarization, and to optimize membrane dynamics, intracellular transport, cell division, and locomotion. In higher eukaryotes, the cytoskeleton is mainly composed of actin microfilaments, intermediate filaments and microtubules (MTs). MTs are dynamic tubulin polymers where the balance between assembly and disassembly of protofilaments is precisely regulated by microtubule-associated proteins (MAPs) [1]. In vertebrates most MTs will disassemble at low temperature but some remain cold-stable or resistant to drugs such as nocodazole [2], [3]. It has been shown that the stability of MTs that are cold- and nocodazole-resistant is largely due to their association with the class of MAP known as MAP6 (previously named STOP for Stable Tubule Only Polypeptide) and MAP6d1 (previously named SL21 for STOP-Like of 21 kD) [2], [4], [5]. MAP6 and MAP6d1 proteins (designated in this paper as MAP6s) are expressed only in vertebrates [6], and have been localized in neurons, astrocytes, oligodendrocytes, fibroblasts, and several tissues (including heart, muscle, lung, and testis) [4], [7], [8], [9]. MAP6 is expressed as three isoforms (MAP6-1, -2, -3) [2]. These MAPs interact with MTs through their MT binding modules called Mn and Mc: MT resistance to both cold and nocodazole disassembly is via the Mn modules, and cold resistance only is via the Mc modules. Interestingly, the Mn modules are well conserved in MAP6 proteins amongst vertebrates, whereas Mc modules are only found in mammals and are the result of a defined insertion in exon 1 [2], [6]. MAP6s modules are bi-functional since MT-stabilizing domains and calmodulin-binding domains overlap to some extent allowing a steric regulation for MT binding as described *in vitro* and *in vivo* [4], [6]. Additionally, when phosphorylated, MAP6-1 does not bind to microtubules *in vitro* and co-localizes with actin filaments *in vivo* suggesting, as for MAP2c (a mammalian neuronal MAP), a role in neurite initiation [10], [11], [12]. MAP6s share a N-terminal cysteine-rich sequence, which, when palmitoylated, targets these proteins to the Golgi apparatus [2], [4]. Map6-null mice exhibit a set of defects similar to those of schizophrenia disorders; thus the loss of Map6 is not lethal but is clearly central for normal synaptic plasticity [13]. Taken together, these data indicate a high level of MAP6s regulation.

*Trypanosoma brucei* (*T. brucei*) is a flagellated protozoan parasite that causes human sleeping sickness and Nagana disease in livestock in central and southern Africa. It belongs to the Kinetoplastidae order of protozoa (characterized by the presence of a kinetoplast, the single mitochondrion genome associated with the base of the flagellum), which includes *Trypanosoma cruzi* and different species of *Leishmania* that are responsible for Chagas disease and leishmaniasis respectively [14]. The *T. brucei* cytoskeleton is composed of a subpellicular corset of about 100 MTs underlying the plasma membrane, the mitotic spindle (MS) in mitotic cells, and a single flagellum [15], [16]. In the flagellum, the MTs form the canonical axoneme with 9 outer triplets of MTs at the basal bodies level followed by the 9 outer doublets at the transition zone and the 9 outer doublets of MTs surrounding a central pair of MTs (9+2) that is characteristic of motile cilia [17].

The flagellum of trypanosomes participates in a wide variety of functions, from cell mobility to host-parasite interaction (for reviews see [18], [19]). It is a highly complex organelle - a proteomic analysis of its cytoskeleton has resulted in the identification of 331 proteins, many of which are conserved in other kinetoplastids and higher eukaryotes [20]. The kinetoplastid flagellum possesses a prominent structure called the paraflagellar rod (PFR), which runs along the axoneme from the point at which the flagellum exits the cell to its distal tip [21] and, which is required for normal flagellum motility [22], [23], [24], [25], [26].

The MT arrays of trypanosomes are highly resistant to depolymerization upon cold treatment [27], [28] and have a low sensitivity to nocodazole [29], [30]. In *T. brucei*, several MAPs have been characterized, but notably none have been described as being flagellar specific [20], [31], [32], [33], [34], [35], [36], [37].

In eukaryotes, the MT-based organelles centrioles, cilia and flagella MT have cold-resistant MTs [38], [39], [40], [41], [42]. The processes regulating cilia and flagella stability are not fully understood but may involve tubulin post-translational modifications [43], [44]. Also, several proteins are involved in their stability such as the ribbon protofilament proteins [45], [46], [47]. The ribbon protofilament protein Rib43a is conserved in trypanosomes [48] and is found in the cytoskeletal fraction of the flagellum, but has not been studied [20], [49], [50].

In this study, we present *Tb*SAXO (for *T. brucei* STOP Axonemal protein) as the first MAP6-related protein identified in a protozoan. We demonstrate that it is an axonemal MAP involved in flagellar motility in *T. brucei*. In addition, our analysis indicates that *Tb*SAXO belongs to a unique group of MAP6-related proteins (SAXO proteins) that are present only in ciliated or flagellated organisms ranging from protozoa to mammals, which suggests for the SAXO proteins unique functions in cilia and flagella of eukaryotes.

## Results

### Identification of MAP6-related proteins from protozoa to mammals

During a proteomic analysis of *T. brucei* flagellar proteins [51], [52], we identified a 30 kD basic protein (GeneDB accession number Tb927.8.6240). Based on the study (described below), we designated this protein as *Tb*SAXO. *Tb*SAXO is listed in a trypanosome flagella proteome analysis as orthologous to an unknown *Plasmodium falciparum* protein (PFI0460w), that we designate here *Pf*SAXO [20].

A BLAST analysis, using the *Tb*SAXO sequence, identified orthologues throughout the taxonomic categories and ranged from protozoa to mammals (Fig. S5). Those orthologs were present only in ciliated or flagellated eukaryotes, with the exception of *Cryptosporidium*, a parasitic non-flagellated, non-ciliated protozoan. Also we were not able to identify orthologues neither in prokaryotes, fungi, nor in the flagellates *Naegleria*, *Phytophthora* and *Monosiga*.

**Figure 1:**
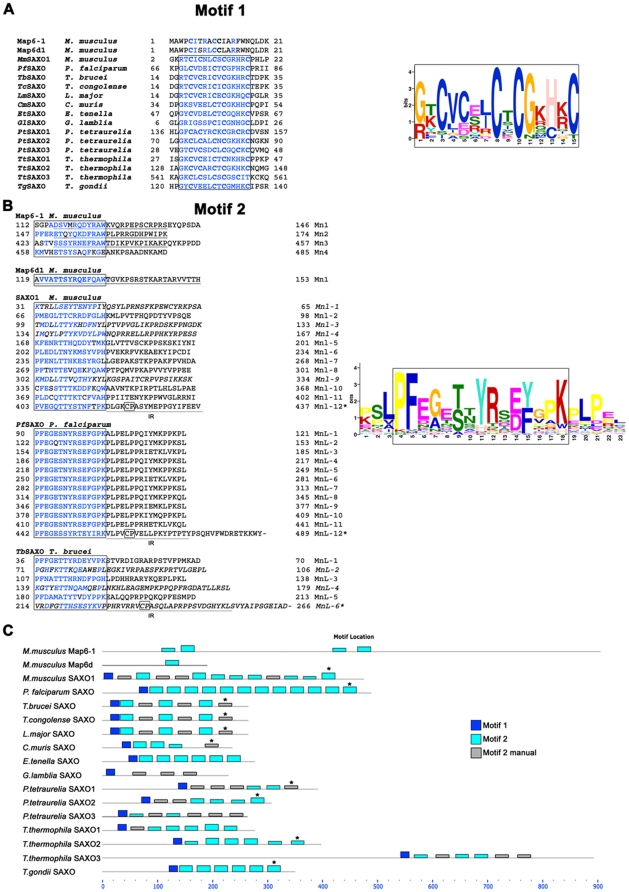
Identification of SAXO proteins, a MAP6-related protein family. **A**. Motif 1. The N-terminal domain and its cysteine consensus sequence. Left panel: alignment of the N-terminal sequences of the proteins used for the MEME analysis in C. The boxed sequences correspond to motif 1. Amino acids corresponding to the regular expression of motif 1 are shown in blue. Right panel: motif 1 is represented as a position-specific probability matrice derived from the MEME analysis in C. **B**. Motif 2. Mn domains in mouse Map6-1, Map6d1, and Mn-like domains and inter-repeats in mouse Saxo1, *Plasmodium* SAXO and *Trypanosoma* SAXO. Left panel: characters in blue correspond to the regular expression of the Mn and Mn-like domains identified by the MEME analysis in C; the Mn-like domains identified manually are in italics. The underlined sequences in Map6-1 and Map6d1 correspond to the experimentally identified Mn domains in mouse [4], [6]. CP motifs are boxed. IR: inter-repeat regions. Right panel: the Mn-like regular expression is represented as position-specific probability matrix derived from MEME analysis in C. **C**. Identification of a family of proteins containing Mn-Like domains. MEME analysis using mouse Map6s, mouse Saxo1, and only protozoan SAXO sequences identified a characteristic N-terminal motif (motif 1, dark blue boxes) in SAXO proteins and Mn-like domains (motif 2, light blue boxes) common to the SAXO and MAP6 proteins. Manually identified supplementary motifs 2 are in grey boxes (Motif 2 manual). The asterisk indicates a conserved CP sequence in the last Mn-like domain.

We analyzed the protein primary sequences of *Tb*SAXO, *Pf*SAXO and *Mm*Saxo1 (one of the two orthologs identified in mouse) and identified four shared amino acid features: 1) a high percentage of proline (13.2%, 20% and 11.6% respectively); 2) a cluster of cysteines at the N-terminus (motif 1); 3) a ∼15 amino acid repeated domain, which is highly conserved in *Pf*SAXO (motif 2) (Fig. 1A, B); and 4) all three SAXO proteins have a basic calculated pI of 8.95, 8.25, 9.05 for *Tb*SAXO, *Pf*SAXO and *Mm*Saxo1 respectively [53]. We did not identify any canonical proline-recognition domains in *Tb*SAXO or *Pf*SAXO, but repeated proline-rich sequences in both proteins as well as several proline-recognition domains (SH2 and SH3 binding domains, data not shown) in *Mm*Saxo1 are indicative of a role in protein-protein interaction [54].

Analysis of the primary sequences of *Mm*Map6s, *Tb*SAXO, *Pf*SAXO and *Mm*Saxo1 indicated that these proteins displayed similar characteristics: 1) a N-terminal domain sequence (motif 1) with a cluster of cysteines (Fig. 1A), and, 2) a repeated domain of 15 amino acids (motif 2) that shared some conserved residues with the MT binding and stabilizing Mn module of MAP6 proteins. The motif 2 is followed by an inter-repeat region (IR) of ∼18–20 residues, which is highly conserved in *Pf*SAXO (Fig. 1B). Additionally, we used the motif analysis tool MEME [55], [56] with mouse Map6-1, Map6d1, *Mm*Saxo1, *Pf*SAXO, *Tb*SAXO and several other protozoan sequences identified by BLAST to further characterize these proteins, in particular the protozoan proteins (Fig. 1C). Keeping in mind that the highly conserved repeat sequence in *Pf*SAXO introduces a strong bias (Fig. 1B), and in spite of this, the analysis identified a regular expression of 2 motifs corresponding to motif 1 ([GR][KT]CVC[ESR][LI]CTCGK[HC][KR]C) and motif 2 ([PK]SLPFE[GA]E[TS][NT]YRS[ED][FY]GPKPLP[EP]L) (Fig. 1A, B). As shown in Figure 1C, motif 1 is present in the protozoan sequences and the *Mm*Saxo1 sequence but absent in Map6-1 and Map6d1 sequences demonstrating its specificity for a distinct group of proteins (SAXO proteins) despite the common presence of cysteines. Motif 2 is repeated and common to all sequences including the mouse Map6s. This latter motif corresponds partially to the MAP6s Mn modules that are involved in MT resistance to both cold and nocodazole depolymerization that were identified experimentally [6] (Fig. 1B). We thus named the regions containing the motif 2 of *Tb*SAXO, *Pf*SAXO and *Mm*Saxo1 Mn-like motifs (MnL). Based on the highly conserved T/S and F/Y residues respectively at positions 6 and 12 of motif 2, we manually identified additional putative MnL domains (Fig. 1B sequences in italic, Fig. 1C motif 2 manual). We identified 6 MnL domains in *Tb*SAXO and 12 domains in *Pf*SAXO and *Mm*Saxo1 (Fig. 1B). The C-terminal MnL domains have a longer sequence than the other MnL domains and bare a short Cysteine-Proline motif (CP). We thus differentiate these domains by an asterisk MnL-x* (Fig. 1B, C). Interestingly, the MEME analysis identifies a fourth Mn (or MnL) domain in Map6-1, which has not been identified previously and which experimentally does not seem to be functional [6].

Taken together, these data suggest that the identified SAXO proteins form a family of MAP6-related proteins in ciliated/flagellated organisms present in protozoa to mammals, and that they are organized as follows: the N-terminus bears a cysteine-rich motif 1 followed by a linear repetition of MnL motifs, the most C-terminal one carrying an additional Cysteine-Proline motif.

### TbSAXO is an axoneme specific protein in the protozoan *T. brucei*

**Figure 2:**
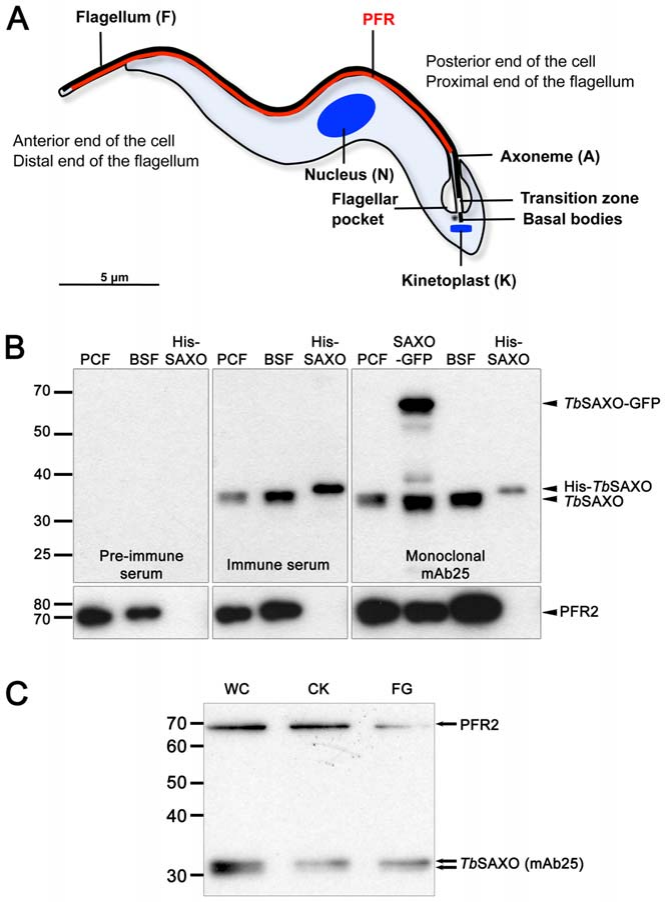
*Tb*SAXO is a cytoskeleton protein in *T. brucei.* **A**. Schematic diagram of a *T. brucei* cell in interphase. (N) nucleus, (K) a single kinetoplast, (F) a single flagellum, (A) a single axoneme, and PFR the single paraflagellar rod. Note that the distal end of the flagellum emerges from the flagellar pocket located at the posterior end of the cell and is then external to the cell body. **B**. Specificity of the polyclonal serum and the monoclonal antibody mAb25 for *Tb*SAXO. Pre-immune and immune sera were tested on 1.10^6^ PCF and BSF cells and 40 ng of the purified recombinant Histidine-tagged *Tb*SAXO (His-SAXO) demonstrating the specificity of the immune serum. The derived monoclonal antibody mAb25 is also specific to *Tb*SAXO as shown on PCF and BSF cells, histidine-tagged recombinant protein as well as PCF cells overexpressing a GFP-tagged *Tb*SAXO (calculated MW 57.3 kD). The loading control was done with the L8C4 antibody, which identifies PFR2 protein. **C**. The monoclonal antibody mAb25 identifies *Tb*SAXO on 3.10^6^ PCF whole cells (WC), cytoskeletons (CK) and flagella (FG). The membranes were probed simultaneously with mAb25 and the loading control anti-PFR2.

During the *T. brucei* cell cycle, the new flagellum originates from the newly formed basal body and grows through the flagellar pocket (see diagram of a *T. brucei* cell, Fig. 2A). At the point of exit from the pocket, the flagellum bares the PFR, an accessory structure that is involved in flagellum motility, and is common to some flagellated protists [21]. In the context of the complex flagellum organization and in order to investigate the location and function of *Tb*SAXO in detail, we cloned the gene and produced a polyclonal and a monoclonal antibody (mAb25). Western-blot (WB) analysis on *T. brucei* whole procyclic form (PCF) cells and bloodstream form (BSF) cells using the polyclonal and mAb25 antibodies showed that the antibodies were specific to *Tb*SAXO and that *Tb*SAXO was expressed in both parasite forms (Fig. 2B). Furthermore, *Tb*SAXO was associated with the cytoskeleton fraction of the flagellum since detergent and salt extraction during the preparation of cytoskeletons and flagella did not remove the protein (Fig. 2C). In addition, WB studies indicated that *Tb*SAXO migrates as a doublet suggesting that one or more post-translational modifications are present on the protein (Fig. 2C).

**Figure 3:**
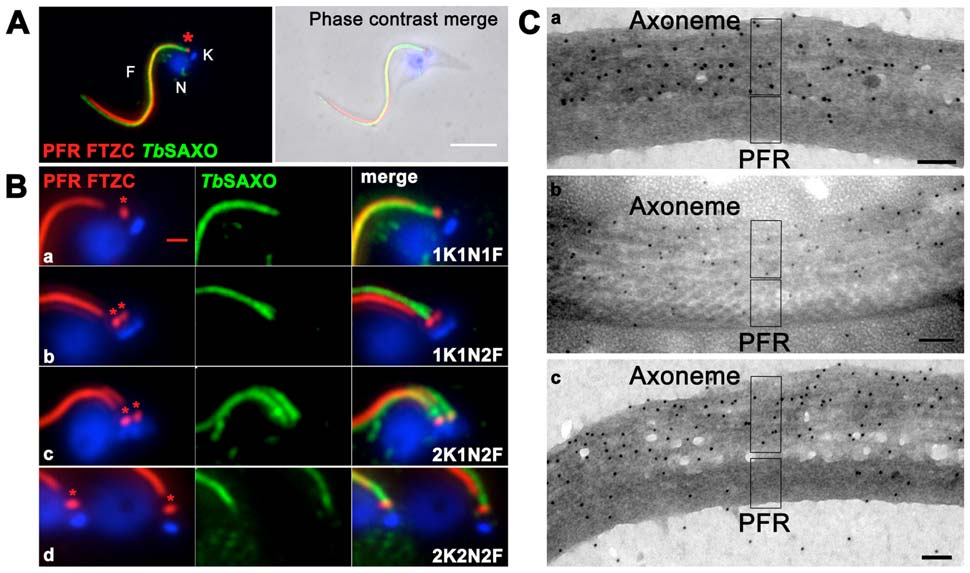
*Tb*SAXO is an axoneme-associated protein in *T. brucei*. **A**. Immuno-labeling and localization of *Tb*SAXO on PCF cytoskeletons. Left panel: *Tb*SAXO localization in the flagellum was identified by the mAb25 antibody (green). Labeling extends along the entire length of the flagellum from the flagellar transition zone (*, labeled with the FTZC antibody) to the distal tip. The PFR is labeled red and begins where the flagellum exits the cell (antibody L8C4). Right panel: a merge of IF and phase contrast. N: nucleus. K: kinetoplast. F: flagellum. Bar, 5 µm. **B**. Images of the proximal flagellar regions of PCF cytoskeletons from cells through mitosis and cytokinesis. In each row, the left panel shows the PFR and FTZC (*) (red), the center panel *Tb*SAXO (green), and the right panel shows merged images. The cell cycle stages are defined as 1K1N1F (1 Kinetoplast, 1 Nucleus, 1 Flagellum), 1K1N2F, 2K1N2F and 2K2N2F in rows a–d respectively. *Tb*SAXO labeling is present immediately distal to the FTZC and is clearly distinct from PFR staining. Bar, 1 µm. **C**. Immuno-gold electron microscopy reveals that *Tb*SAXO is localized in the axoneme. Mab25 immuno-gold particles can be seen mainly on the axoneme and not on the PFR of flagella of PCF WT cells. Bars, 100 nm.

In both PCF and BSF developmental stages, *Tb*SAXO was observed by immuno-fluorescence (IF) to localize along the flagellum (Fig. 3 for PCF, Fig. S4 for BSF). Fluorescence signal extended from the transition zone of the axoneme to the distal tip of the flagellum as shown by the co-labeling with the well-established transition zone antibody FTZC [57] and the anti-PFR2 antibody L8C4 [58] (Fig. 3A, B). *Tb*SAXO was also present in both the old and new flagella in dividing cells (Fig. 3B and Fig. S4). The flagellar signal was visible even when the short, new flagellum was entirely within the flagellar pocket; this was confirmed by the absence of PFR labeling on the new flagellum (Fig. 3B, 1K1N2F). Double labeling of the PFR and *Tb*SAXO suggested that *Tb*SAXO is an axoneme-associated protein, in that *Tb*SAXO localization extends past the PFR, is proximal to the transition zone, and there was no over-lap or co-localization along the length of the flagellum beside some parts of the flagellum due to the orientation of the cell when observed by immuno-fluorescence (Fig. 3A, B). Further, immuno-electron labeling of PCF flagella revealed mAb25 labeling all over the axoneme (Fig. 3Ca, b, c). Unfortunately, the stronger fixation conditions required for whole cell preparation prevented immuno-labeling of sectioned material.

### TbSAXO is a microtubule-associated and microtubule-stabilizing protein

We have shown that *Tb*SAXO is an axoneme-associated protein in the parasite with primary sequence characteristics similar to those of the MAP6s; thus we asked the question could it function in MT binding and/or stabilization?

**Figure 4:**
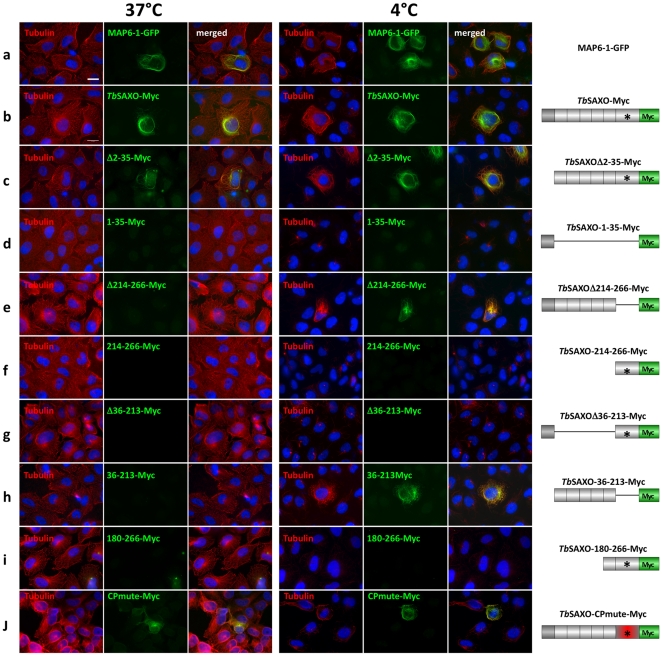
*Tb*SAXO is a microtubule-associated protein and a microtubule-stabilizing protein. Mammalian cells (U-2 OS) expressing either MAP6-1-GFP (row a), *Tb*SAXO-Myc (row b), or various truncated versions of *Tb*SAXO-Myc (rows c–j) (constructs are represented on the schemes on the right panel). In each case, the transfected cells were incubated at 37°C or 4°C to test for MTs cold stability. Anti-tubulin (TAT1) and anti-Myc antibodies provided the images in left and centre columns respectively at each temperature. The right columns for each temperature set are merged images. The cells were subjected to short extraction before fixation and immuno-labeling. *Tb*SAXO MT stabilization is seen in images b, c, e, h and j. MT stabilization is also observed in the positive control MAP6-1-GFP expressing cells (a). Nuclei were labeled with DAPI. Bar, 20 µm.

To study *Tb*SAXO MT stabilizing functions directly, and to circumvent the fact that the trypanosome cytoskeleton is unusually stable [20], [27], [29], [30], we expressed a recombinant *Tb*SAXO-Myc protein in the U-2 OS human cell line (human bone osteosarcoma epithelial cells) [59]. As with many mammalian cell lines, when U-2 OS cells in culture are incubated either at 4°C or in the presence of nocodazole, interphase MTs depolymerize (Fig. S1A, B). To validate the use of the U-2 OS cell system for our purposes, we transfected them with a vector allowing the expression of the rat MAP6-1-GFP protein. After 24 h post-transfection, the cells were briefly detergent-extracted then fixed. At 37°C MAP6-1-GFP was expressed in U-2 OS cells and decorated the interphase MTs (Fig. 4a). After cold or nocodazole treatment, only cells expressing MAP6-1-GFP displayed a network of MTs, demonstrating that the MAP6 protein had stabilized the MTs (Fig. 4a, Fig. S1b).

We next transfected the cells with the *Tb*SAXO-Myc expression vector to study its potential to stabilize MTs. At 24 h post transfection, the cells were extracted and fixed and *Tb*SAXO-Myc was detected by immuno-staining using an anti-Myc antibody. At 37°C, *Tb*SAXO-Myc decorated the MT network (Fig. 4b), similar to what we had observed with MAP6-1-GFP transfection demonstrating that *Tb*SAXO can function as a microtubule-associated protein in a heterologous system, and can interact with interphase MTs at 37°C. We never observed *Tb*SAXO-Myc labeling on the mitotic spindle or at the midbody, suggesting some specificity for interphase MTs in this system. After cold (4°C) treatment, we observed the depolymerization of MTs in non-transfected cells as would be expected, but in cells expressing *Tb*SAXO-Myc, stabilized MTs were observed to be decorated with *Tb*SAXO-Myc protein (Fig. 4b). In summary, these data show that *Tb*SAXO can act as a MT binding and stabilizing protein.

### The MnL motifs are required and sufficient for microtubule binding and stabilization

One of the characteristics of MAP6s is the presence of a N-terminal cysteine cluster, which can be palmitoylated and is responsible for targeting to the Golgi apparatus [4]. We wanted to determine if the N-terminus of *Tb*SAXO was also involved in targeting *per se*; thus, we first deleted the N-terminal domain (*Tb*SAXOΔ2-35-Myc). When *Tb*SAXOΔ2-35-Myc was expressed in U-2 OS cells, it co-localized with MTs at 37°C (Fig. 4c [extracted cells] and Fig. S2c [non-extracted cells]), and stabilized the MT network at 4°C (Fig. 4c) suggesting that the N-terminal domain of *Tb*SAXO is not required for MT targeting and binding. Further, the N-terminal domain alone was not sufficient for MT binding since *Tb*SAXO-1-35-Myc did not bind to MTs at 37°C or 4°C, and there was no MT stabilization at 4°C (Fig. 4d), although expression of the recombinant protein was confirmed in non-extracted cells (Fig. S2d). Finally, none of the full-length (*Tb*SAXO-Myc), the N-terminal domain (1–35-Myc) and the N-terminal truncation (Δ2-35-Myc) proteins co-localized with Giantin (a Golgi marker) in non-extracted cells. Thus, we can conclude that the N-terminal domain is not involved in Golgi targeting in this system (Fig. S6).

In *Tb*SAXO, we identified 6 potential MnL domains (Fig. 1B). We thus investigated the MT-stabilizing properties of the repetitions MnL-1 to MnL-5 (as a block) and the MnL-6* domain (which is longer than the other MnL domains and carries the CP motif) by preparing Myc-tagged constructs and transfecting U-2 OS cells. The C-terminal domain deletion construct (ΔMnL-6*, *Tb*SAXOΔ214-266-Myc) did not co-localize with the MT network at 37°C but co-localized with stabilized MTs after 4°C treatment (Fig. 4e). It was thus clear that cold treatment induces a re-localization of the recombinant protein, as previously observed for Map6-3 in NIH3T3 cells [2]. The MnL6* domain (*Tb*SAXO-214-266-Myc) alone was not sufficient for MT targeting and or binding and stabilization since MnL-6* was not associated with the cytoskeleton in either experimental condition (Fig. 4f) although expression of the recombinant protein was confirmed in non-extracted cells (Fig. S2f).

Finally, we deleted the MnL-1 to MnL-5 motif block (*Tb*SAXOΔ36-213-Myc) and observed that there was no association of this expressed protein with MTs at either 37°C or 4°C (Fig. 4g and Fig. S2g). Notably, expression of the block of MnL-1 to MnL-5 modules (*Tb*SAXO-36-213-Myc) was able to restore the MT targeting/binding and stabilizing functions after 4°C treatment suggesting a cold-induced re-localization (Fig. 4h). The cold-induced relocalization occurred only in the absence of the MnL6* domain (*Tb*SAXOΔ214-266-Myc and *Tb*SAXO-36-213-Myc) suggesting a role of this domain in MT-binding at 37°C. Since MnL6* carries a specific Cysteine-Proline motif, we mutated the CP motif to Glycine-Asparagine and showed that the mutation did not affect the MT-binding and stabilizing properties of *Tb*SAXO-Myc (compare b and j in Fig. 4 and Figure S2). This data shows that the CP motif is not involved in MT binding at 37°C and that another signal of unknown nature might be involved.

Full-length or N-terminal deleted *Tb*SAXO-dependent stabilization of MTs was achieved in nocodazole treated cells but required the MnL6 motif since the construct 36–213-Myc did not stabilize microtubules after nocodazole treatment (Fig. S1B). Further, mutation of the CP motif in MnL6* did not modify the MT binding or stabilizing properties of *Tb*SAXO after nocodazole treatment (Fig. S1Bg), suggesting that this motif is not involved in the nocodazole-stabilizing property of the protein.

Finally, whilst being expressed in U-2 OS cells, the MnL6* domain associated to MnL5 (180–266-Myc) was not able to bind MT neither at 37°C nor at 4°C and did not induce cold or nocodazole MT-stabilization suggesting that more than two MnL domains are necessary (Fig. 4i, S1Bf and S2i).

These data demonstrate that the MnL motifs of *Tb*SAXO do share functional properties with the Mn domains of known MAP6 proteins. MnL6* and MnL5-MnL6* domains are not sufficient for MT targeting/binding or stabilization but modules MnL-1 to MnL-5 are sufficient for MT targeting/binding and stabilization after cold treatment. This suggests that, unlike Map6d1 which has only one Mn module to stabilize MTs, more than two MnL modules might be required for MT binding and stabilization in *Tb*SAXO.

### The MnL-1-5 domains are required for flagella targeting/binding in *T. brucei*

**Figure 5:**
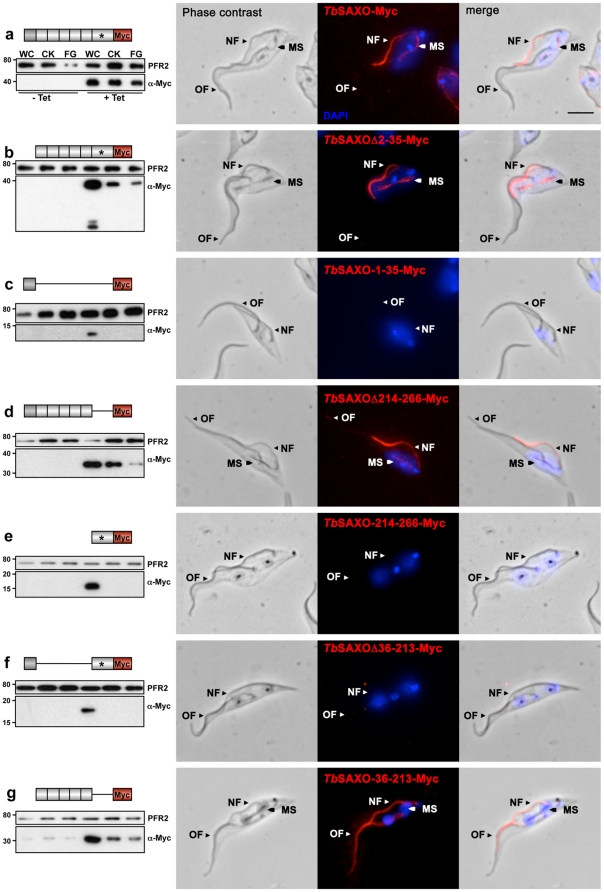
The MnL domains are required for flagellum targeting and binding in *T. brucei*. Immuno-localization using WB (left panels) and IF (right panels) of C-terminal, Myc-tagged, *Tb*SAXO truncations after over-expression in PCF cells. WB panels: 3.10^6^ whole cells (WC), cytoskeletons (CK) and flagella (FG) were loaded on a 15% SDS-PAGE gel. The membranes were probed simultaneously with L8C4 (for PFR loading control) and anti-Myc antibodies. Lanes 1–3: non-induced cells (−Tet). Lane 4–6: cells induced for over-expression (+Tet) for 24 h, except *Tb*SAXO-1-35-Myc (c), which was induced for 48 h. Immuno-fluorescence panels: cytoskeletons from over-expressing cells were probed with anti-Myc antibody. The kinetoplasts and nuclei were labeled with DAPI. Expression of the Myc-recombinant proteins was induced for 6 h except for *Tb*SAXO-36-213-Myc (g), which was induced for 18 h. OF: old flagellum. NF: new flagellum. MS: mitotic spindle. Nuclei and kinetoplasts were labeled with DAPI. These results indicate that the Mn domains are necessary and sufficient for targeting *Tb*SAXO to the flagellum. Bar, 5 µm.

Because of the high stability of trypanosome MTs, the MT stabilization properties of *Tb*SAXO could not be directly assessed in the parasite, but, we investigated the domain(s) involved in flagellum targeting and binding. We transfected procyclic trypanosomes with an over-expression vector allowing the inducible expression of various *Tb*SAXO-Myc truncations of the protein (schematically represented in Fig. 5). For all the transfected cell lines studied, none of the trypanosome (non-induced or induced) developed any growth phenotypes when compared to the non-transfected parental cell line (WT) (data not shown). All Myc-constructs were expressed upon induction as shown by WB using whole cells probed with anti-Myc antibody (Fig. 5) and by IF (Fig. S3). Immuno-fluorescence experiments on cytoskeleton-extracted cells (Fig. 5) and on whole cells (Fig. S3) showed that, for all constructs, a pool of Myc-tagged protein was found to be soluble, localized in the cytoplasm, and was extracted during the cytoskeleton preparations.

As shown on the WB in Figure 5a, the intensity of the full-length over-expressed protein is only slightly reduced in induced flagella compared to whole cells and cytoskeletons suggesting that most of the recombinant protein is targeted to and associated with the flagellum. After a short induction time of expression of recombinant protein (3–6 h), the tagged *Tb*SAXO was directed to the new but not the old flagellum (Fig. 5a, IF panel) suggesting a slow turnover of the protein or cell cycle dependent targeting. Also, a weak IF signal was observed on the mitotic spindle (MS). This labeling was never observed on WT cytoskeletons probed with mAb25 but was also observed for over-expressed *Tb*SAXO-GFP (unpublished data) suggesting that *Tb*SAXO-Myc is targeted to the newly made flagellum, and that the MS targeting may be an artifact of over-expression. Different expression levels were observed between the truncated forms of recombinant *Tb*SAXO-Myc, as shown on WB (for example Fig. 5b versus 5c). Thus, it was not possible to directly compare the flagellar MT affinity of these proteins when analyzing their expression profiles in whole cells, cytoskeletons or flagella.

Our IF and WB data revealed that the MnL-1 to MnL-5 motif block (*Tb*SAXO-36-213-Myc) was necessary and sufficient to target and to bind to the flagellum since its deletion (*Tb*SAXOΔ36-213-Myc) abolished the axoneme and the MT location of the protein suggesting that the targeting/binding domain has been removed (Fig. 5f, g). Further neither the N-terminal motif 1 (*Tb*SAXO-1-35-Myc) nor the MnL-6* domain (*Tb*SAXO-214-266-Myc) were necessary for flagellum targeting or binding since their tagged version were soluble and their deletion (*Tb*SAXOΔ2-35-Myc, *Tb*SAXOΔ214-266-Myc) did not affect the axoneme location of the protein (Fig. 5b–e, Fig. S3). Since *in vivo* expression of a small recombinant protein might be difficult (*Tb*SAXO-1-35-Myc is 10.2 kD), we expressed a C-terminus GFP-tagged version (*Tb*SAXO-1-35-GFP, 30.6 kD). Again, the motif 1-GFP construct was produced as a soluble pool (unpublished data). In contrast to *Tb*SAXOΔ2-35-Myc and *Tb*SAXOΔ214-266-Myc, we did not detect any *Tb*SAXO-36-213-Myc labeling at the MS, even after longer induction times when both old and new flagella were decorated (Fig. 5g, IF).

Taken together, these data demonstrate that the MnL domains are required for targeting/binding to the flagellum but that neither the MnL-6* nor motif 1, carrying the cysteine cluster, is required for flagellum targeting or binding. Moreover, these findings support the importance of multiple MnL domains for the MT targeting that we have observed for *Tb*SAXO in mammalian cells (Fig. 4).

### TbSAXO is involved in flagellum motility in *T. brucei*

**Figure 6:**
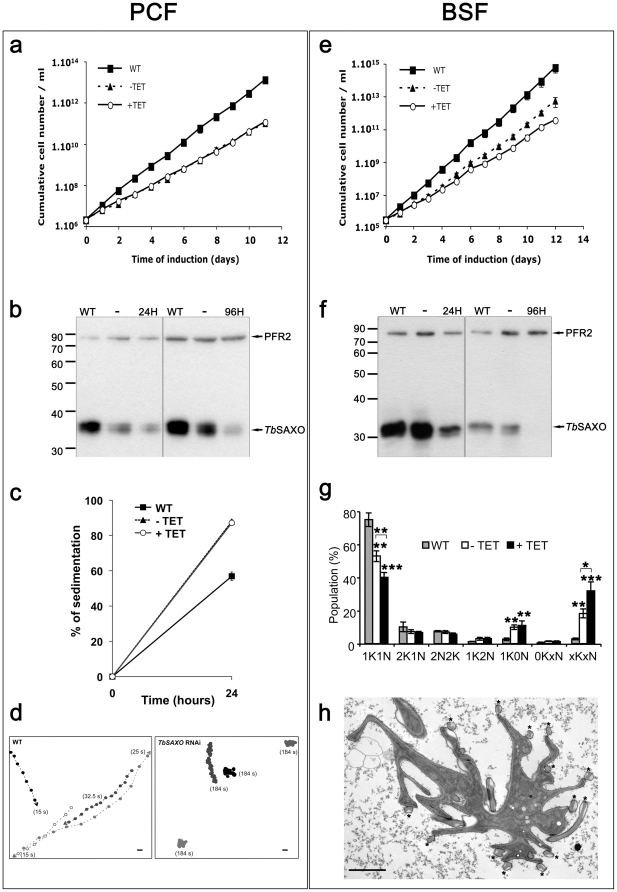
*Tb*SAXO RNAi knockdowns exhibit impaired flagellar motility. Inducible RNAi*^TbSAXO^* in PCF (**a, b, c, d**) and BSF (**e, f, g, h**) cells. Growth curves of PCF (**a**) and BSF (**e**) RNAi*^TbSAXO^* cell lines. Corresponding WBs (PCF in **b**, BSF in **f**) of WT (parental), RNAi non-induced (-), and 24 h and 96 h induced cells probed with mAb25 and L8C4 (anti-PFR2). For PCF 5.10^6^ cells were used and 1.25×10^5^ cells for BSF. **c**. Sedimentation assay of PCF RNAi. WT (closed squares). RNAi non-induced (−TET) (closed triangles) and induced (+TET) (open circles). **d**. Mobility graph obtained from Movie S1. The positions of individual cells are plotted at 2.5 s intervals. Open circle: starting position of each cell. Arrowhead: ending position. Number in parentheses: time in seconds of a given cell was within the field of view. Bar, 10 µm. **g**. Graph of cell populations with orthodox and unorthodox kinetoplast number in BSF RNAi cultures (72 h of induction). K: kinetoplast. N: nucleus. Asterisks indicate statistical significance compared with the WT population, and −TET *versus* +TET condition (*P<0.1; ** *P<0.05*; ***P<0.01). **h** Electron-micrograph of a thin section of an aberrant BSF RNAi induced cell (72 h). (*) indicates a flagellum. Scale bar, 2 µm. Error bars in a, c, e, and g represent the standard error from 3 independent experiments.

To assess the functional role of *Tb*SAXO in the parasite, we used the tetracycline-inducible RNA interference (RNAi) system in PCF and BSF cells (kind gifts from G. Cross, Rockefeller University) [57], [60], [61], [62]. Overall, a reduced growth rate was observed for both non-induced PCF and BSF cell lines transformed by the RNAi constructs compared to the non-transformed parental cell line (WT) indicating that there is an induction in the absence of tetracycline or, in other words, a leaky induction of RNAi (Fig. 6a, e). Taking this into account, there was no difference in growth rate observed in induced PCF cells compared to non-induced cells (Fig. 6a). WB analysis of the *Tb*SAXO expression level showed that RNAi induction strongly reduced the expression of *Tb*SAXO in PCF while some reduction of expression was also observed in non-induced cells when compared to WT cells (Fig. 6b). This occurred in numerous clones and after numerous transformations. In BSF cells, induction of RNAi also strongly reduced the expression of *Tb*SAXO (Fig. 6f), some cells retained a very weak *Tb*SAXO IF labeling after 72 h of RNAi knockdown (multinucleate and anucleate cells in Fig. S4B), but this is possibly the consequence of cell death within the 72 h induction period and thus the absence of continued RNAi knockdown.

Both life-cycle stages displayed different RNAi*^TbSAXO^* phenotypes but their flagella had consistent length and morphology indicating that flagellar MTs were not destabilized when *Tb*SAXO expression is reduced. *TbSAXO* RNAi is not lethal in PCF cells, however cell mobility was reduced since cells sedimented suggesting a flagellar motility defect (Fig. 6c). Exemplified by mobility traces (Fig. 6d) and supported by Movie S1, we confirmed the motility defect by illustrating that the RNAi*^TbSAXO^* cells remained primarily in one location (<0.4 µm/sec) whilst the WT cells travelled long distances (>10 µm/sec). The RNAi*^TbSAXO^* cells were not paralyzed, but the flagellar beat appeared uncoordinated and slower when compared to WT (Movie S2). Also, a small proportion of the PCF cells (5%) were either zoids (1 kinetoplast, no nucleus) or multi-flagellated (4 flagella) indicating some difficulties in cytokinesis when *Tb*SAXO protein level was reduced (Fig. S4A).

Cytokinesis defects have previously been described as a consequence of RNAi knock-down of several proteins involved in flagellar motility in BSF trypanosomes, such as PFR2, TAX-1 and Trypanin, resulting in cells with multiple nuclei and kinetoplasts [20], [63]. Even though induction of *TbSAXO* RNAi in BSF was not unequivocally lethal, non-induced cells displayed a slight reduction in growth rate, which was further accentuated in induced cells (Fig. 6e). Reduction of the expression of *Tb*SAXO in BSF cells (Fig. 6f) induced a cytokinesis-defective phenotype that resulted in an accumulation of cells with aberrant numbers of kinetoplasts, nuclei and flagella (Fig. 6g and Fig. S4B). Non-induced (leaky) and induced cells initiated multiple S phase and mitosis cycles but appeared to lack subsequent cytokinesis, or in some cases underwent incorrect cytokinesis, leading to accumulation of multinucleated cells and anucleated cells (zoids). The multinucleated and multi-flagellated BSF cells (xKxNxF) had a highly convoluted cell periphery and accumulation of vesicles of unknown composition as observed by electron microscopy on thin-sections (Fig. 6h). These aberrant cells are unlikely to recover to the normal phenotype.

To summarize, these data demonstrate that *Tb*SAXO is involved in flagellum motility in PCF and as a consequence in cytokinesis in both forms of *T. brucei*.

## Discussion

In this study, we have identified *Tb*SAXO as an axoneme-specific MAP protein in the protozoan *T. brucei*. By analyzing its primary sequence and performing functional analysis, we have demonstrated that this protein has the characteristics of a structural MAP. Further as discussed in detail below, identification of a cysteine-rich N-terminal domain (motif 1), and of the Mn-like domains (motif 2) involved in stabilization of MTs upon cold and nocodazole treatments, suggests that *Tb*SAXO is the first MAP6-related protein to be identified in protozoa. *Tb*SAXO co-localizes with MTs (in both the parasite and mammalian cells), and when expressed in mammalian cells, it stabilizes microtubules and induces MT bundles as is observed for the expression of higher eukaryotic structural MAPs in heterologous cells [64], [65], [66], [67].

Knockdown of *Tb*SAXO using RNAi*^TbSAXO^* in its natural context reduced flagellar motility suggesting that *Tb*SAXO plays a role in flagellar motility. The phenotypes induced by the RNAi*^TbSAXO^* (flagellar motility defect in PCF and cytokinesis defect in BSF) are not unique; knock-down of other proteins involved in flagellum motility in *T. brucei* [20], [63], [68] also produce these phenotypes, even though Ralston et al. demonstrated recently that normal flagellum motility is not required in BSF [69]. Although flagellum motility (in PCF) and cytokinesis (in BSF) are compromised in the RNAi*^TbSAXO^* cell lines, no defect in flagellum biogenesis, structure or MT stabilization was observed. This suggests that some *Tb*SAXO that remains after RNAi*^TbSAXO^* knockdown is sufficient, or that other protein(s) can compensate for reduced levels of *Tb*SAXO. *Tb*SAXO may have more than one function in trypanosomes. In mammalian cells, we have observed that *Tb*SAXO has intrinsic microtubule stabilization properties as is the case for two other unrelated *T. brucei* MAPs (CAP15 and CAP17) [36]. This suggests that *Tb*SAXO may also contribute to MT rigidity in the flagellum.

The structural MAPs Tau and Map2 have 3–4 repeats of ∼30 amino acids in the microtubule-binding domain, each repeat binding to tubulin. They stabilize microtubules by binding longitudinally individual protofilaments [70], [71]. Binding of Tau or Map2c induces an increase of MT rigidity by changing the mechanical properties of the polymer *in vitro* [72], [73]. Our observations suggest a similar mechanism for *Tb*SAXO in flagellar beat or flagellar motility in trypanosome. Our immuno-localization and functional studies suggest that in the wild-type flagellum *Tb*SAXO binds to the protofilaments of outer doublets microtubules, and modulates the rigidity of the polymer thus influencing the beating properties of the flagellum. When *Tb*SAXO level is decreased (by RNAi), the flagellum beating properties may change and are directly reflected by the motility phenotype observed in PCF. From our IF studies, *Tb*SAXO co-localizes with the 9+2 region of the axoneme (distal to the transition zone and continues to the distal tip of the flagellum). The central pair of MTs and associated proteins (also named the central apparatus) is responsible for the motility of the axoneme in motile cilia and flagella [74]. Modulation of the beating properties of the flagellum via *Tb*SAXO is thus coherent with its localization. *Tb*SAXO could therefore bind microtubules to modify or impose rigidity rather than cold resistance. Additionally, the consequences of *Tb*SAXO RNAi may induce compensational modulation of flagellum beat or stability by other proteins, and or may influence the role of the PFR in motility; thus identifying the exact role of *Tb*SAXO cannot be defined without considering the functions of structural and stabilizing proteins and the changes made after *Tb*SAXO knockdown.

In general, MT-based functions are regulated by the recruitment of MAPs via regions carrying specific tubulin post-translational modifications. In trypanosomes, tubulins are highly post-translationally modified. In particular, acetylated tubulin was identified in the flagellum and subpellicular MTs in *T. cruzi* [75] and in the flagellum of *T. brucei* [76]. Also, these MTs are glutamylated and tyrosinated but not glycylated [77], [78], [79]. By expressing different domains of *Tb*SAXO in mammalian cells and in *T. brucei*, we have identified the MnL domains as necessary for targeting, binding and stabilizing MTs and for targeting to the flagellum. Since *Tb*SAXO is a MAP, the post-translational status of tubulins might be the “signal” or “code” required for *Tb*SAXO targeting to the microtubules of the axoneme and not the microtubules of the cellular corset. Although other processes such as intra-flagellar transport (IFT) could also be involved [80], even though IFT has been recently demonstrated not to be specific to ciliated/flagellated cells [81].

*Tb*SAXO and the MAP6 protein share an N-terminal domain carrying a cluster of cysteines, the Mn (or Mn-Like) modules, and cold and nocodazole MT stabilizing property suggesting that *Tb*SAXO is a Map6-related protein. However, the differences in the sequence of the IR regions, the number of repetitions of MnL modules, and the absence of a strict consensus sequence for the N-terminal domain suggest some functional differences. When palmitoylated at the N-terminus, some MAP6s will localize to the Golgi, although their exact function there is not clear [4]. *Tb*SAXO was not observed at the Golgi complex in *T. brucei* nor U-2 OS cells (our study) and was not identified in a *T. brucei* protein palmitoylation analysis [82] suggesting a lack of palmitoylation. What could be the role of the N-terminal domain of *Tb*SAXO? When phosphorylated, the structural MAPs such as tau, MAP2, MAP4 and MAP6-1 detach from MTs and can interact with other proteins such as actin [10], [83], [84], [85]. The *Tb*SAXO doublet band observed in our western-blots could reflect phosphorylation of *Tb*SAXO in both PCF and BSF. This is supported by the phosphorylations observed at the N-terminus (serine 8, serine 9 and tyrosine 12) in the phospho-proteome of BSF [86]. There is no direct evidence of a MT-unbound form of *Tb*SAXO in our data but like other MAPs, *Tb*SAXO could have a modified MT-binding profile or structure in the phosphorylated state thus modulating the rigidity of the MTs in response to extracellular signals. Additionally, the presence of several proline-recognition domains in *Tb*SAXO could suggest interactions with other flagellar proteins [54].

Until now MAP6 proteins have only been identified in vertebrate cells. Axoneme localization and a role in flagellum motility have not been described to date for any of the MAP6 proteins. Our databases/literature searches identified SAXO orthologs from protozoan to mammals but only in ciliated/flagellated organisms, with the exception of *Cryptosporidium*. A specific role for SAXO proteins in cilia and flagella is strongly suggested since proteomes of flagella of sperm cells (in rat, mouse and drosophila), flagella of *T. brucei*, *Chlamydomonas* and cilia of human airway cells have SAXO orthologs [20], [87], [88], [89], [90], [91]. A SAXO ortholog was identified in a total extract of *C. elegans*, which has ciliated sensory neurons [92]. In contrast, no SAXO orthologs was listed in the mouse photoreceptor neuron proteome. Map6 does appear in samples containing the cytoskeleton rootlet but not in the sample containing only the axoneme [93]. Taken together this points to a specific role for SAXO proteins in flagella and cilia function. We predict that data generated from additional analyses would clearly open new avenues in the study of this protein family, and its role in axonemal function and diseases.

## Materials and Methods

### Ethics statement

All animal experiments were performed in accordance with institutional guidelines as determined by the Service Commun des Animaleries de l'Université Bordeaux Segalen (approval number A33-063-916), which specifically approved the study. All experiments requiring genetically modified organisms were carried out with approval of the ethics committee of the Ministère de l'Enseignement Supérieur et de la Recherche (approval number 4946).

### In silico analysis

SAXO orthologues were identified by walking BLASTP (BLAST 2.2.26 [94], [95], [96]) using default parameters (non redundant protein sequences database, BLOSUM 62 matrix, gap cots existence: 11; extension: 1; conditional compositional score matrix adjustment, no filter, no mask) and a cut-off E-value of 4e-06 using initially *Tb*SAXO or *Pf*SAXO as query. Identified proteins were then also used as query proteins.

Settings for MEME analysis [55] were: any number of repetition, optimum width of each motif of 6 minimum and 30 maximum, 2 maximum motifs. Accession numbers of protein sequences used for the MEME analysis are: *Mus musculus* Map6-1 (NP_034967.2), *Mus musculus* Map6d1 (NP_941001.2), *Mus musculus* Saxo1 (NP_001074565.1), *Plasmodium falciparum Pf*SAXO (PFI0460w), *Cryptosporidium muris* (XP_002141332.1), *Eimeria tenella* (SNAP00000003849), *Giardia lamblia* (EFD95462.1), *Paramecium tetraurelia*_1 (XP_001444884.1), *Paramecium tetraurelia*_2 (XP_001446307.1), *Paramecium tetraurelia*_3 (XP_001430086.1), *Tetrahymena thermophila*_1 (XP_001030650.1), *Tetrahymena thermophila*_2 (XP_001026633.1), *Tetrahymena thermophila*_3 (XP_001025437.1), *Toxoplasma gondii* (XP_002365452.1), *Trypanosoma brucei brucei Tb*SAXO (XP_847454.1, Tb927.8.6240), *Trypanosoma cruzi* (XP_817934.1), *Trypanosoma congolense* (TcIL3000.8.6080), *Leishmania major* (XP_001683716.1).

### Statistical analysis

In all experiments, error bars represent standard deviation (SE) of samples in triplicates. Statistics in Figure 6g were performed using an unpaired two-tailed *t*-test with a 90%, 95% or 99% confidence interval. XLSTAT software (XLSTAT.com) was used to determine *P*-values.

### Cell lines, growth conditions and transfection

Genomic DNA of the *T. brucei* cell line TREU927/4 GUTat10.1 (a kind gift from S. Melville, Cambridge University) selected by the genome project [97] was used to amplify by PCR the ORFs of the proteins studied here. The work described in this study uses the parental PCF and BSF *T. brucei* 427 strains (kind gifts from G. Cross, Rockefeller University), co-expressing the T7 RNA polymerase and a tetracycline repressor, named in this study as wild-type (WT) [98]. Over-expression and RNAi were induced with 1 µg.mL^−1^ tetracycline. PCF trypanosomes (*T. brucei* 427 29–13) were grown and transfected as in [99]. BSF trypanosomes (*T. brucei* 427 90–13) were grown and transfected as in [100]. Special care was taken to not keep in culture the selected PCF and BSF transformants more than 2 weeks because of revertion over long-term culture [101]. Transformants were screened by immuno-fluorescence, after tetracycline induction and cloned by serial dilution. Correct integration in the PCF mini chromosomes (MCs) of the pPRP-177 vector was checked by pulsed-field electrophoresis and southern-blot as described in [62], [102]. Growth curves were done by using a mallassez cell counter every 24 h and by diluting the PCF cells back to 2.10^6^ cells/ml and the BSF back to 3.10^5^ cell/ml. Growth curves in Figure 6 represent the cumulative cell number.

U-2 OS cells (human bone osteosarcoma epithelial cells, ATCC® Number: HTB-96, [103]) were grown in D-MEM Glutamax (Gibco) supplemented with 10% fetal calf serum and 1% Penicillin-Streptomycin at 37°C plus 5% CO_2_. Exponentially growing cells were transfected with 1 µg DNA using Lipofectamine 2000 in OPTIMEM (Invitrogen) according to the manufacturer's instructions and processed for IF 24 h post-transfection.

### Plasmid construction

The *TbSAXO* ORF was amplified from *T. brucei* 927 genomic DNA using the two specific primers EcoRI-1019 (5′-CCCGAATTCATGACAACATTGCACACTATTTCCAGCC-3′) and 1019-HindIII (5′-AAAAAAGCTTCTAATCCGCAATTTCTCCTGAGGGG-3′) and cloned into pET28a+ (Novagen) in frame with a N-terminal 6-histidine-tag making the pDRET1 plasmid used for bacterial expression and purification. To produce the *TbSAXO* RNAi cell lines (PCF and BSF), a 650 bp insert was amplified by PCR (primers 600PRP-XbaIBamHI 5′-TTCAGAGGGATCCCTCGAGTCTAGAAAGTCACGAACGTCGGGCATCGATT-3′ and HindIII-1019 5′-AAAAGTTCGAAGCTTATGACAACATTGCACACTATTTCCAGCC-3′) and cloned into the p2T7-177 plasmid [62], [102] thus providing pPRP-177. We have checked the pPRP-177 vector by sequencing the 177 pb repeat sequences, the promoter regions and the RNAi target sequence. The *Not*I linearized plasmid was transfected in PCF and BSF cells. For the expression of Myc-tagged *Tb*SAXO, the full-length *TbSAXO* ORF lacking the stop codon was cloned into the *Hind*III-*Xba*I sites of pLew100X-Myc vector containing a C-terminal 3×Myc-tag and of a pLew100X-GFP plasmid containing a C-terminal GFP (pLew100 vector from [98] modified in the lab). For generation of truncated versions of *Tb*SAXO, PCR amplification of the desired *TbSAXO* sequence was performed to yield fragments that encode aa 1–35, 36–213, 214–266, and deletion of aa 2–35, 36–213 and 214–266. For deletion of aa 36–213, an overlapping PCR was made using as template the fragment encoding 1 to 35 aa and the fragment encoding aa 214 to 266 of *Tb*SAXO. The PCR products were cloned into the *Hind*III-*Xba*I sites of the aforementioned vectors and verified by sequencing. To make the mammalian expression vectors for the *Tb*SAXO-Myc constructs, all Myc-tag ORFs were amplified by PCR from the original pLew100X-Myc constructs (including the stop-codon down-stream the 3×Myc tag) and cloned directly into the pcDNA3.1/CT-GFP TOPO mammalian (Invitrogen). Fused domains MnL5-MnL6-Myc (aa 180–266-Myc) was amplified from *TbSAXO-Myc* full-length in the pcDNA3.1/CT-GFP vector and cloned into pcDNA3.1/CT-GFP. Mutation of the Cysteine-Proline motif in MnL6* (C_236_P_237_ into Glycine_236_-Alanine_237_) was done by site-directed mutagenesis following the instruction from the Quickchange Site-directed mutagenesis Stratagene kit. All DNA sequences were checked by DNA sequencing.

### Bacterial expression and purification of 6His-TbSAXO

The BL21(*DE3*) (Novagen) bacterial strain, transformed with pDRET1, was grown in 200 mL of LB+kanamycin (50 mg.mL^−1^) to an OD_600 nm_ of 0.5. Expression of the recombinant protein was induced by adding 1 mM IPTG for 3 hours at 37°C. Cells were harvested and resuspended in 20 mL 50 mM NaPi pH 7.0, 150 mM NaCl, then lysed by sonication. Cell debris and inclusion bodies were pelleted by centrifugation (4500× g) for 30 min at 4°C. The pellet was resuspended in 20 mL 50 mM NaPi pH 7.2, 50 mM NaCl (buffer B), sonicated and washed twice in buffer B. The recovered pellet was solubilized in 5× buffer B plus 8 M Urea. The 6His-*Tb*SAXO protein was purified on a 1.25 mL HIS-Select™ Cartridge (Sigma) Nickel affinity gel eluted with a gradient of Imidazole (0 mM–250 mM) in buffer B plus 8 M urea. The chosen fractions were pooled and dialyzed against buffer B plus 8 M urea at 4°C.

### Production of the anti-TbSAXO monoclonal antibody

100 µg of 6His-*Tb*SAXO was emulsified with complete Freund's adjuvant (for first injection) or incomplete Freund's adjuvant (following boosts) and used to immunize two Balb/C female mice (Charles River Laboratories, L'Arbresle, France). Spleen cells of one mouse were fused to myeloma X63-Ag8 cells as in [104]. One hybridoma producing a *Tb*SAXO-specific antibody was selected and named mAb25 (IgG2a).

### *T. brucei* flagellar protein preparation

Flagellar proteins were prepared, separated in denaturing conditions on a BIORAD ROTOFOR (ampholins 3–10) followed by SDS-PAGE electrophoresis as in [52]. After Coomassie staining, two protein bands were excised, trypsin-digested and subjected to LC-MS/MS.

### Western blots

Proteins were prepared in Laemmli buffer [105] separated by SDS-PAGE (10, 12 or 15% acrylamide) and transferred onto PVDF membranes. Membranes were processed as in [99]. After washes in Tris buffer saline (TBS), 0.2% Tween-20 then TBS, membranes were washed in TBS and revealed with 0.05% diaminobenzidine, 0.015% H_2_O_2_ or ECL (Immobilon Millipore) according to the manufacturer's instructions. The primary antibodies used were the anti-*Tb*SAXO monoclonal mAb25 (1∶1000), anti-PFR2 L8C4 (1∶1000), anti-cMyc 1∶200 (Santa Cruz SC-40), and the secondary antibody was an IgG+IgM HRP-conjugated 1∶10,000 (Jackson, 115-035-044).

### Sedimentation assays and video-microscopy

Sedimentation assays were done as in [23]. Briefly, WT, non-induced (−tetracycline) and induced (+tetracycline) RNAi*^TbSAXO^* cells were grown for 24 h then placed in cuvettes. The OD_600 nm_ was measured at t = 0 and t = 24 h before (ODb) and after (ODa) mixing. The graph represents the percentage of sedimentation calculated as 100-(ODb/ODa ×100). Video-microscopy was carried out as described in [106]. Briefly, PCF cells (WT cell and RNAi*^TbSAXO^* cells after 72 h of induction) were washed in PBS and flagellum motility and cell mobility were recorded by phase contrast on a Zeiss AxioImager, 100× lens (NA 1.4) and 40× (NA 1.3) respectively. 35 s or 184 s of digital video from separate regions were captured and analyzed using Metamorph® software (Molecular Devices).

### Immuno-fluorescence

#### *T. brucei* cells

*T. brucei* PCF cells were treated as in [52] and fixed in paraformaldehyde 3% in PBS. BSF cytoskeletons were prepared by washing the cells in vPBS (PBS, sucrose 15.7 g.L^−1^, glucose 1.8 g.L^−1^), then gently resuspending in PIPES 100 mM pH 6.9, MgCl_2_ 1 mM, NP-40 0.25% and directly loading them on poly-L-lysine-coated slides and finally fixed in methanol (−20°C, 30 min). After rehydration in PBS the slides were incubated 1 hour at room temperature with primary antibodies in a dark moist chamber followed by two washes (5 min) in PBS then incubation in the secondary antibody (1 h). After two 5 min washes in PBS, DNA was labeled 5 min with DAPI (10 µg.mL^−1^) followed by 2 PBS washes. Slides were mounted with Slowfade® Gold Kit (Molecular Probes, S-36936). Combination labeling was as follows: for localization of the *Tb*SAXO Myc-tag proteins we used monoclonal anti-Myc 1∶20 (Santa-cruz sc-40) and anti-mouse IgG (H+L) Alexafluor-594 conjugated (Molecular probes A21201, 1∶400); for triple labeling (*Tb*SAXO, PFR and flagellar transition zone), we used simultaneously mAb25 1∶5 (IgG2a); anti-PFR2 neat (L8C4, IgG1), and anti-transition zone FTZC 1∶10,000 (rabbit polyclonal). Secondary antibodies used were: anti-mouse IgG2a specific Alexafluor-488 conjugated (Molecular probes, A-21131, 1∶400), anti-mouse IgG1 specific Alexafluor-594 conjugated (Molecular probes, A-21125, 1∶400) and anti-rabbit IgG (H+L) Alexafluor-594 (Molecular probes, A-11012, 1∶400). It is noteworthy that the IF studies of *Tb*SAXO-Myc constructs were done after short induction times (6 h, except *Tb*SAXO-36-213-Myc which was induced 18 h), to avoid cellular saturation with recombinant proteins.

#### U-2 OS cells

For the MT stability assays in U-2 OS cells at 37°C and 4°C (cells were incubated 30 min at 4°C), all solutions were pre-warmed at 37°C or cooled at 4°C, including the fixation step; alternatively cells were incubated in medium supplemented with 20 µM nocodazole 30 min, 37°C, to induce MT depolymerization. For observation of whole cells, cells grown on coverslips were washed briefly with 30 µl of EMT buffer (PIPES 60 mM, HEPES 25 mM, EGTA 10 mM, MgCl_2_ 10 mM adjusted to pH 6.9 with KOH) and fixed in paraformaldehyde 3% in EMT for 15 minutes (at 37°C or 4°C). To remove soluble proteins (including non-MT associated proteins), cells were briefly extracted for 2 min with 30 µl of EMT, TX-100 0.5%, glycerol 10% then fixed in paraformaldehyde (at 37°C or 4°C, 15 min). Cells were neutralized 10 min in glycine (100 mM in PBS). After two washes in PBS, cells were incubated in PB (PBS, 10% fetal calf serum, 0.01% saponin) for 10 minutes. Primary antibodies anti-cMyc (rabbit polyclonal Sigma c-3956, 1∶100) and anti-tubulin (TAT1, 1∶100, [107]) were added for 1 hour in a dark moist chamber. After two PBS washes, cells were incubated for 1 hour with the secondary antibodies: anti-rabbit FITC conjugated (sigma F-9887, 1∶400) and anti-mouse IgG (H+L) Alexafluor-647 conjugated (Molecular probes, A-21235, 1∶400) respectively. The nuclei were stained with DAPI (1 µg.mL^−1^ in PBS for 5 minutes) and cells were washed and mounted as described above.

Images were acquired on a Zeiss Imager Z1 microscope, using a Photometrics Coolsnap HQ2 camera, at 100× or 63× (NA 1.4) with Metamorph® software (Molecular Devices), and processed with ImageJ.

### Electron microscopy and immuno-electron microscopy

Cells were prepared as in [99] with the following changes: 50 mL of log phase BSF WT and RNAi*^TbSAXO^* cells (72 hours induction) were used. Cells were fixed in 25 mL of 4% glutaraldehyde, 4% paraformaldehyde, and 0.5% tannic acid, in 0.1 M cacodylate buffer pH 7.4. Immuno-labeling of flagella was done as follows. Flagella were prepared as in [52] and resuspended at 1.7×10^7^ flagella/µL. Ten µL of purified flagella were mixed with 100 µL of PBS and place on a sheet of clean Parafilm. Charged Butvar and carbon-coated nickel grids (EMS G200-Ni) were floated onto the droplet for 15 minutes. The flagella were fixed by transferring the grids to 500 µl of 2% Paraformaldehyde, 0.025% Glutaraldehyde in PBS for 30 min. Grids were then neutralized by transferring to 500 µl droplets of 100 mM Glycine in PBS 2×10 min. Grids were transferred to 30 µL of mAb25 (neat) for 4 hours. The grids were washed (three times 10 minutes) in PBS and then incubated on 30 µL drop of 10 nm gold conjugate protein A (Aurion) (diluted 1∶10 in PBS, overnight at 4°C). Grids were then washed 4×10 min in PBS, then fixed 30 minutes in 100 µL droplets of 2.5% glutaraldehyde in distilled water, washed 4×5 min on 500 µL droplets of water, and negatively stained (NanoVan, Nanoprobes; 40 µL grid).

## Supporting Information

Figure S1 **The U-2 OS cell line is a valid model for the cold- and nocodazole-induced MT depolymerization test.** The MTs of U-2 OS cells were labeled with anti-tubulin and nuclei with DAPI. **A**. IF on mock transfected U2-OS cells subjected to 37°C/4°C treatment and a short extraction before fixation. **B**. MAP6-1-GFP (b), *Tb*SAXO-Myc (c) or various truncated versions (d–f) and a mutated version of *Tb*SAXO-Myc (g) was expressed in U-2 OS cells and tested by IF after nocodazole treatment. In each panel, the last columns are merged images. Scale bars represent 20 µm.
(TIF)

Figure S2 **IF on U-2 OS whole cells expressing** ***Tb*** **SAXO-Myc truncations or MAP6-1-GFP.** Experimental conditions were as in Figure 4 except that the cells were fixed before permeabilization in order to visualize the soluble pool of the recombinant proteins and to demonstrate their expression. In each temperature regime, the left column shows tubulin (red), the center column shows the recombinant protein (green), and the right column the merged images. Scale bar represents 20 µm.
(TIF)

Figure S3 **IF on** ***T. brucei*** **whole cells expressing** ***Tb*** **SAXO-Myc truncations.** Experimental conditions were as in Figure 5 except that the cells were fixed before permeabilization to visualize the soluble pool of the recombinant proteins and demonstrate their expression. Scale bar represents 5 µm.
(TIF)

Figure S4 **IF of** ***Tb*** **SAXO RNAi cells lines.** PCF (**A**) and BSF (**B**) cytoskeletons of WT, non-ind (NInd) and Induced (Ind) cells probed by IF with mAb25 and L8C4. **A. a to r**: *Tb*SAXO labelling with mAb25. **a′ to r′**: PFR2 labelling with L8C4. **a–c**: 2K2N wild-type cell. **d–f**: Non induced 2K2N cell showing that mAb25 labeling is partial. **g–i**: 24 h induced 2K2N cell. **j–l**: 48 h induced 2K2N cell. **m–o**: 48 h induced 2K1N multi-flagellated cell. **p–r**: 48 h induced 2 K zoid cell. **a′–c′**: 2K2N wild type cell. **d′–f′**: Non induced 2K2N cell. **g′–i′**: 24 h induced 2K1N cell. **j′–l′**: 24 h induced 2K1N cell. **m′–o′**: 48 h induced xKxN cell. **p′–r′**: 24 h induced 1 K zoid cell. **B. a to r**: *Tb*SAXO labeling with mAb25. **a′ to r′**: PFR2 labeling with L8C4. **a–c**: 2K2N wild type cell. **d–f**: Non induced 2K2N cell showing that mAb25 labelling is weaker than in wild-type cells. **g–i**: 24 h induced 2K2N cell showing that the new flagellum is not labelled. **j–l**: 24 h induced 2K2N cell. **m–o**: 72 h induced multi-flagellated cell showing that some flagella are not labelled. **p–r**: 72 h induced 1 K zoid cell. **a′–c′**: 2K2N wild type cell. **d′–f′**: Non induced 2K1N cell. **g′–i′**: 24 h induced 2K2N cell. **j′–l′**: 24 h induced 4K2N cell. **m′–o′**: 24 h induced xKxN cell. **p′–r′**: 24 h induced 2 K zoid cell. Scale bar represents 5 µm.
(TIF)

Figure S5 **SAXO orthologues were identified from protozoa to mammals.** Settings for MEME analysis were: any number of repetition, optimum width of each motif of 6 minimum and 30 maximum, 2 maximum motifs. Accession numbers of proteins used for the MEME analysis are: *Mus musculus* Map6-1 (NP_034967.2), *Mus musculus* Map6d1 (NP_941001.2), *Gallus gallus* MAP6 (NP_990250.1), *Xenopus tropicalis* MAP6 (NP_001120222.1), *Dano rerio* MAP6 (XP_002664657.1), *Mus musculus* SAXO1 (NP_001074565.1), *Gallus gallus* SAXO1 (XP_424824.1), *Xenopus tropicalis* SAXO (NP_998882.1), *Dano rerio* SAXO (NP_001032667.1), *Trichoplax adhaerens* SAXO (XP_002114668.1), *Drosophila melanogaster* SAXO (NP_650706), *Strongylocentrotus purpuratus* SAXO (XP_787867.1), *Ciona intestinalis* SAXO (XP_002127009.1), *Caenorharbditis elegans* SAXO (NP_492403, T08G11.3), *Nematostella vectensis* SAXO (XP_001640941.1), *Physcomitrella patens* SAXO1 (XP_001761920.1), *Physcomitrella patens* SAXO2 (XP_001774535.1), *Chlamydomonas reinhardtii* (XP_001697232.1, FAP257), *Paramecium tetraurelia* SAXO1 (XP_001444884.1), *Paramecium tetraurelia* SAXO2 (XP_001427791.1), *Paramecium tetraurelia* SAXO3 (XP_001430086), *Cryptosporidium muris* SAXO (XP_002141332.1), *Plasmodium falciparum* SAXO (XP_001351967.1, PFI0460W), *Trypanosoma brucei* SAXO (XP_847454.1, Tb927.8.6240), *Leishmania major* SAXO (XP_001683716.1). **A**. MEME analysis, including SAXO orthologues from protozoa to mammals but also MAP6 proteins from different vertebrates, identified the motif 1 (in red), motif 2a (dark blue), and motif 2b (light blue). **B**. Position-specific probability matrix derived from the MEME analysis for motif 1, motif 2a and motif 2b and their respective regular expression. In this wide-range analysis, the initial motif 2 identified in Figure 1B is, here, identified as 2 divergent motifs (motif 2a and 2b) but where the sequence PFEGE[TS][TN]Y[RK]S[DE][FY]GP[KW] is well conserved (in red).
(TIF)

Figure S6 **The N-terminus domain of** ***Tb*** **SAXO is not involved in Golgi targeting.** Immuno-fluorescence on U-2 OS cells not transfected (a) or expressing (in green, labelled with anti-Myc) *Tb*SAXO-Myc (b), the N-terminus (1–35)-Myc (c) or the deletion construct Δ2-35-Myc (d) co-stained with the Golgi marker anti-Giantin (in red) [108]. Nuclear DNA is labeled with DAPI. Bar, 8 µm.
(TIF)

Movie S1 Illustration of cell mobility of WT cells followed by RNAi induced cells (72 h) confirming the flagellum motility defect of the RNAi*^TbSAXO^* cells, which remained primarily in one location whilst the WT cells travelled long distances.
(MOV)

Movie S2 Illustration of the flagellar beating of a WT cell followed by a 72 h RNAi*^TbSAXO^* induced cell showing that RNAi*^TbSAXO^* cells were not paralyzed, but the flagellar beat appeared uncoordinated, and slower when compared to WT.
(MOV)
