# Supplementary figures and images for: A MAP6-Related Protein Is Present in Protozoa and Is Involved in Flagellum Motility
Source: PLoS One. 2012 Feb 15;7(2):e31344. doi: 10.1371/journal.pone.0031344 (PMC3280300; doi:10.1371/journal.pone.0031344)

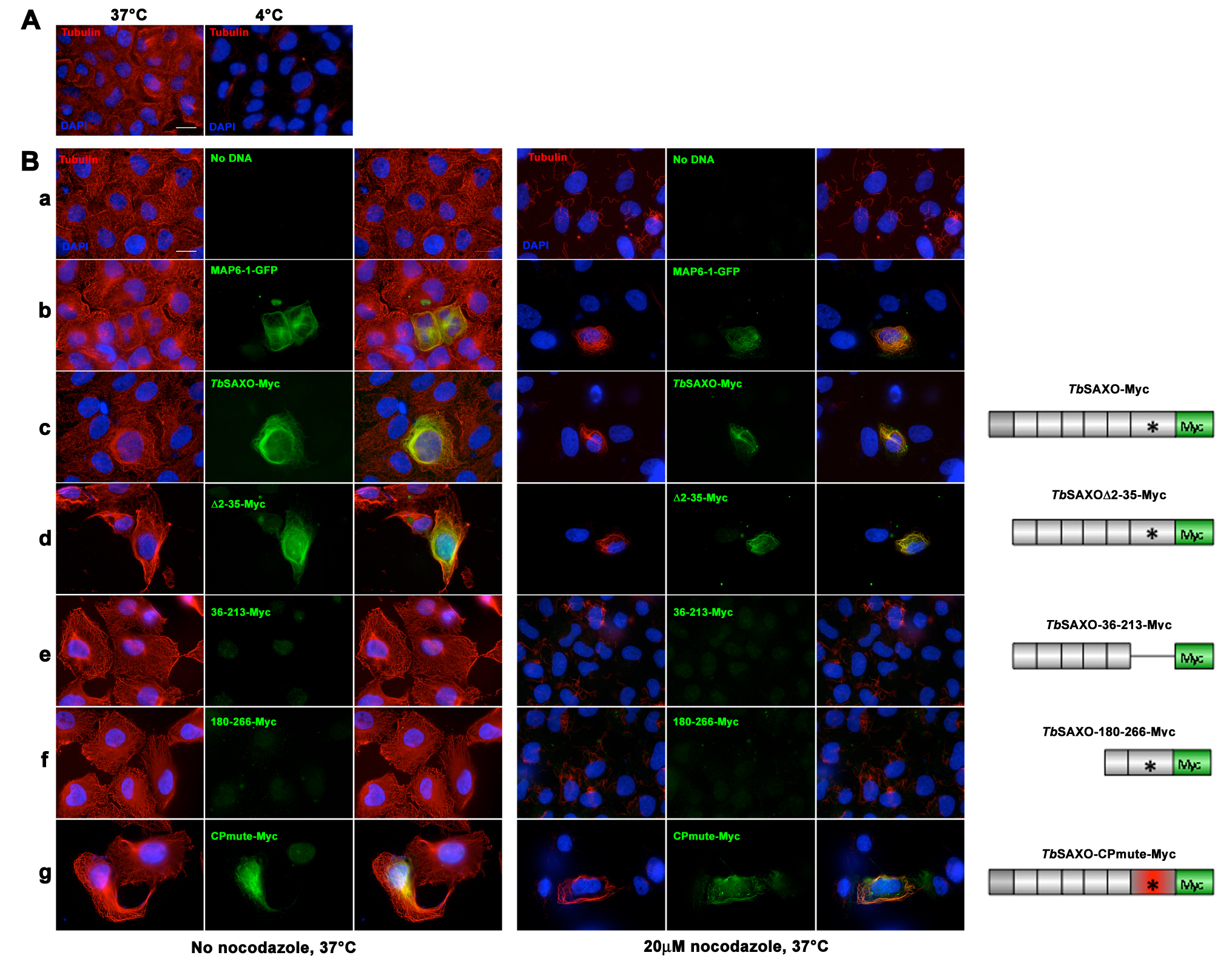

Supplement: Figure S1 — The U-2 OS cell line is a valid model for the cold- and nocodazole-induced MT depolymerization test. The MTs of U-2 OS cells were labeled with anti-tubulin and nuclei with DAPI. A. IF on mock transfected U2-OS cells subjected to 37°C/4°C treatment and a short extraction before fixation. B. MAP6-1-GFP (b), TbSAXO-Myc (c) or various truncated versions (d–f) and a mutated version of TbSAXO-Myc (g) was expressed in U-2 OS cells and tested by IF after nocodazole treatment. In each panel, the last columns are merged images. Scale bars represent 20 µm. (TIF) [file pone.0031344.s001.tif]

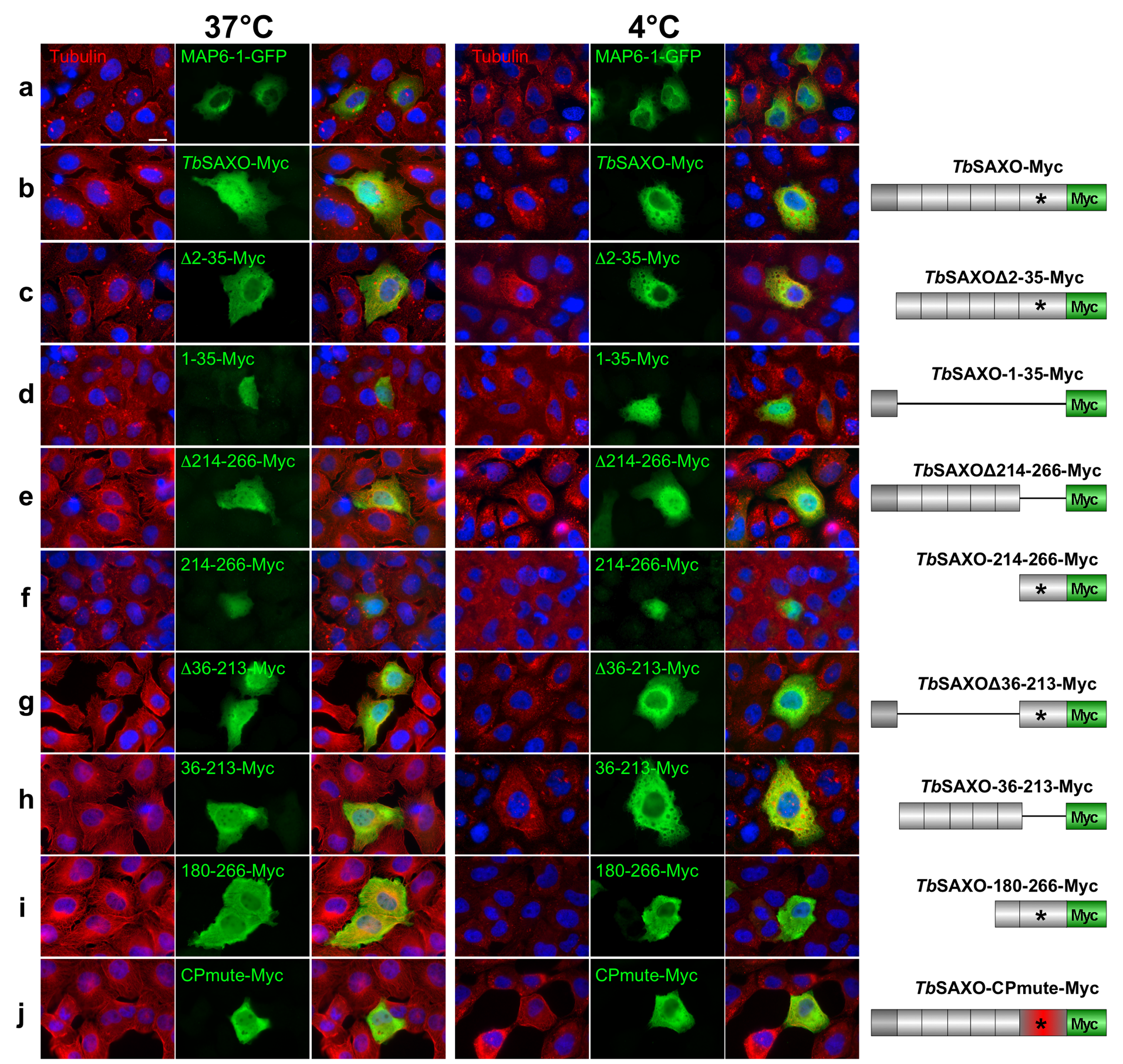

Supplement: Figure S2 — IF on U-2 OS whole cells expressing Tb SAXO-Myc truncations or MAP6-1-GFP. Experimental conditions were as in Figure 4 except that the cells were fixed before permeabilization in order to visualize the soluble pool of the recombinant proteins and to demonstrate their expression. In each temperature regime, the left column shows tubulin (red), the center column shows the recombinant protein (green), and the right column the merged images. Scale bar represents 20 µm. (TIF) [file pone.0031344.s002.tif]

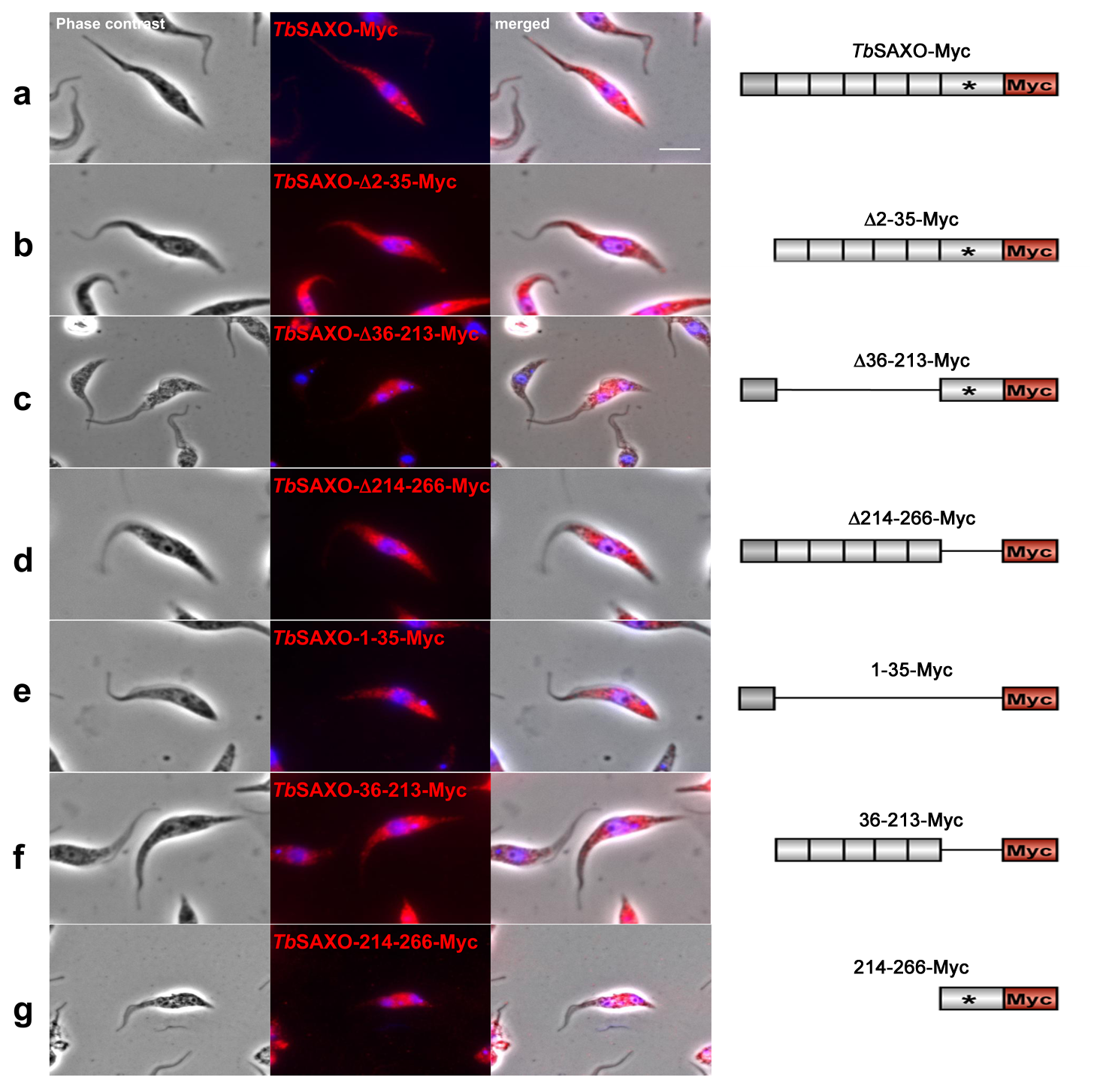

Supplement: Figure S3 — IF on T. brucei whole cells expressing Tb SAXO-Myc truncations. Experimental conditions were as in Figure 5 except that the cells were fixed before permeabilization to visualize the soluble pool of the recombinant proteins and demonstrate their expression. Scale bar represents 5 µm. (TIF) [file pone.0031344.s003.tif]

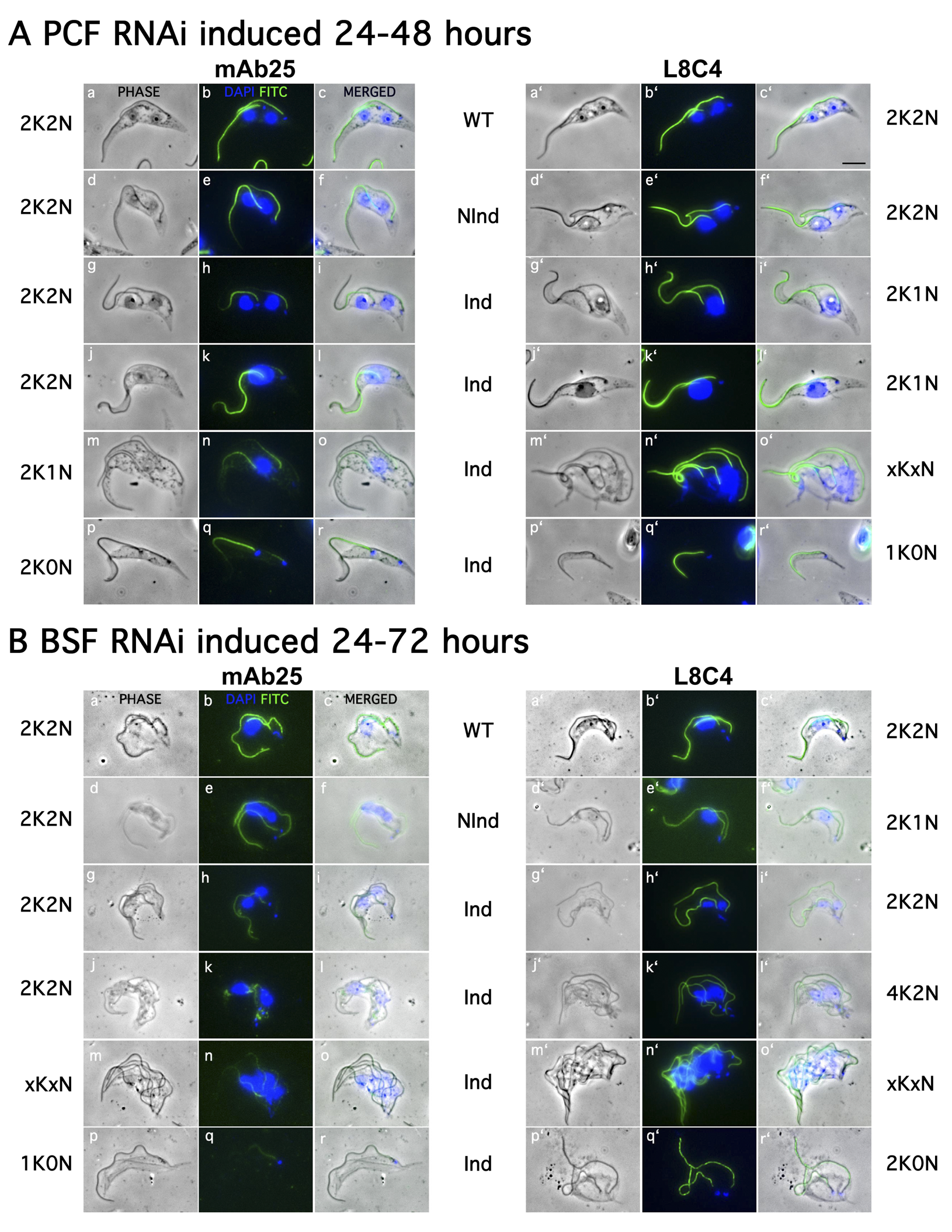

Supplement: Figure S4 — IF of Tb SAXO RNAi cells lines. PCF (A) and BSF (B) cytoskeletons of WT, non-ind (NInd) and Induced (Ind) cells probed by IF with mAb25 and L8C4. A. a to r: TbSAXO labelling with mAb25. a′ to r′: PFR2 labelling with L8C4. a–c: 2K2N wild-type cell. d–f: Non induced 2K2N cell showing that mAb25 labeling is partial. g–i: 24 h induced 2K2N cell. j–l: 48 h induced 2K2N cell. m–o: 48 h induced 2K1N multi-flagellated cell. p–r: 48 h induced 2 K zoid cell. a′–c′: 2K2N wild type cell. d′–f′: Non induced 2K2N cell. g′–i′: 24 h induced 2K1N cell. j′–l′: 24 h induced 2K1N cell. m′–o′: 48 h induced xKxN cell. p′–r′: 24 h induced 1 K zoid cell. B. a to r: TbSAXO labeling with mAb25. a′ to r′: PFR2 labeling with L8C4. a–c: 2K2N wild type cell. d–f: Non induced 2K2N cell showing that mAb25 labelling is weaker than in wild-type cells. g–i: 24 h induced 2K2N cell showing that the new flagellum is not labelled. j–l: 24 h induced 2K2N cell. m–o: 72 h induced multi-flagellated cell showing that some flagella are not labelled. p–r: 72 h induced 1 K zoid cell. a′–c′: 2K2N wild type cell. d′–f′: Non induced 2K1N cell. g′–i′: 24 h induced 2K2N cell. j′–l′: 24 h induced 4K2N cell. m′–o′: 24 h induced xKxN cell. p′–r′: 24 h induced 2 K zoid cell. Scale bar represents 5 µm. (TIF) [file pone.0031344.s004.tif]

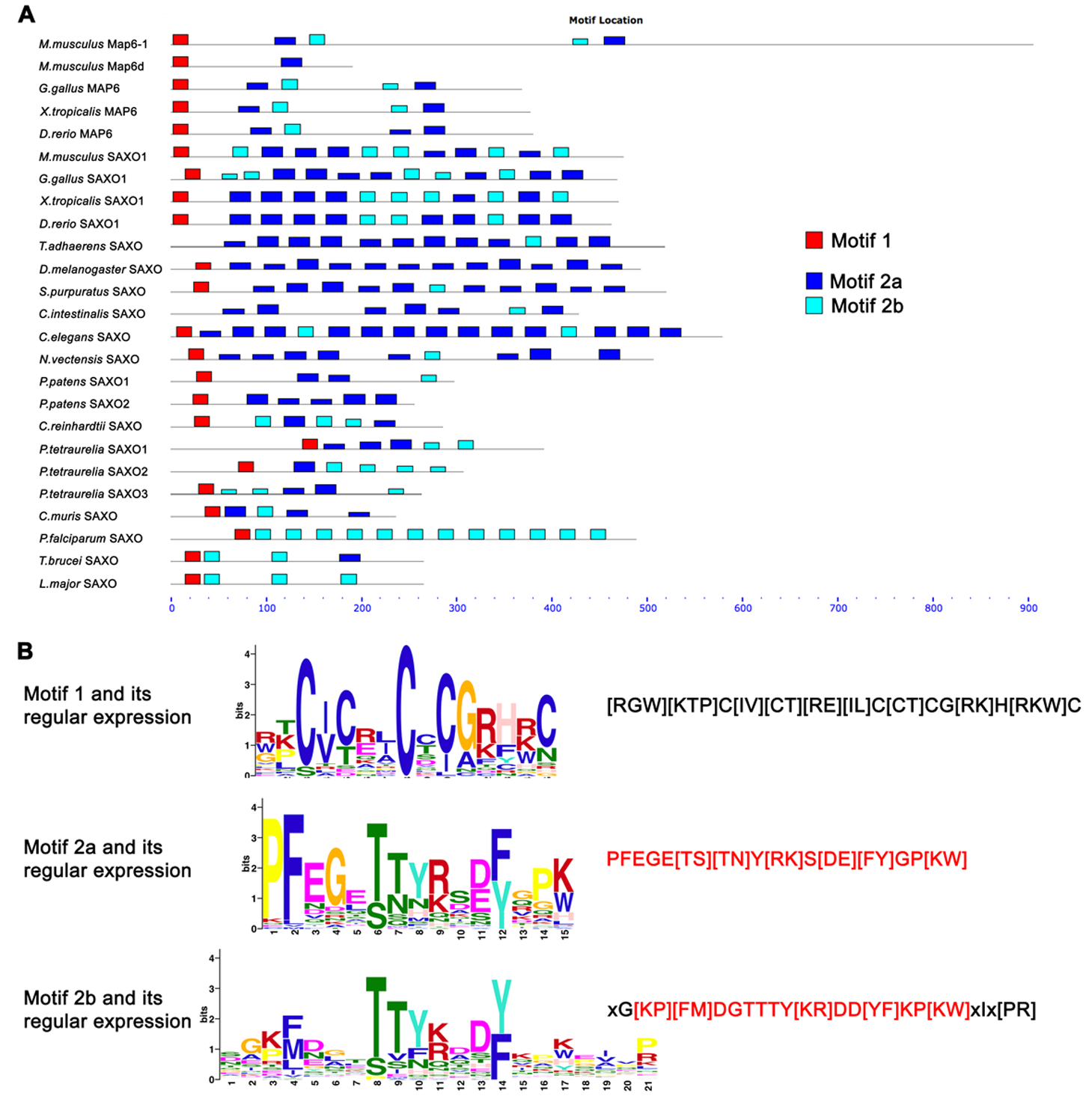

Supplement: Figure S5 — SAXO orthologues were identified from protozoa to mammals. Settings for MEME analysis were: any number of repetition, optimum width of each motif of 6 minimum and 30 maximum, 2 maximum motifs. Accession numbers of proteins used for the MEME analysis are: Mus musculus Map6-1 (NP_034967.2), Mus musculus Map6d1 (NP_941001.2), Gallus gallus MAP6 (NP_990250.1), Xenopus tropicalis MAP6 (NP_001120222.1), Dano rerio MAP6 (XP_002664657.1), Mus musculus SAXO1 (NP_001074565.1), Gallus gallus SAXO1 (XP_424824.1), Xenopus tropicalis SAXO (NP_998882.1), Dano rerio SAXO (NP_001032667.1), Trichoplax adhaerens SAXO (XP_002114668.1), Drosophila melanogaster SAXO (NP_650706), Strongylocentrotus purpuratus SAXO (XP_787867.1), Ciona intestinalis SAXO (XP_002127009.1), Caenorharbditis elegans SAXO (NP_492403, T08G11.3), Nematostella vectensis SAXO (XP_001640941.1), Physcomitrella patens SAXO1 (XP_001761920.1), Physcomitrella patens SAXO2 (XP_001774535.1), Chlamydomonas reinhardtii (XP_001697232.1, FAP257), Paramecium tetraurelia SAXO1 (XP_001444884.1), Paramecium tetraurelia SAXO2 (XP_001427791.1), Paramecium tetraurelia SAXO3 (XP_001430086), Cryptosporidium muris SAXO (XP_002141332.1), Plasmodium falciparum SAXO (XP_001351967.1, PFI0460W), Trypanosoma brucei SAXO (XP_847454.1, Tb927.8.6240), Leishmania major SAXO (XP_001683716.1). A. MEME analysis, including SAXO orthologues from protozoa to mammals but also MAP6 proteins from different vertebrates, identified the motif 1 (in red), motif 2a (dark blue), and motif 2b (light blue). B. Position-specific probability matrix derived from the MEME analysis for motif 1, motif 2a and motif 2b and their respective regular expression. In this wide-range analysis, the initial motif 2 identified in Figure 1B is, here, identified as 2 divergent motifs (motif 2a and 2b) but where the sequence PFEGE[TS][TN]Y[RK]S[DE][FY]GP[KW] is well conserved (in red). (TIF) [file pone.0031344.s005.tif]

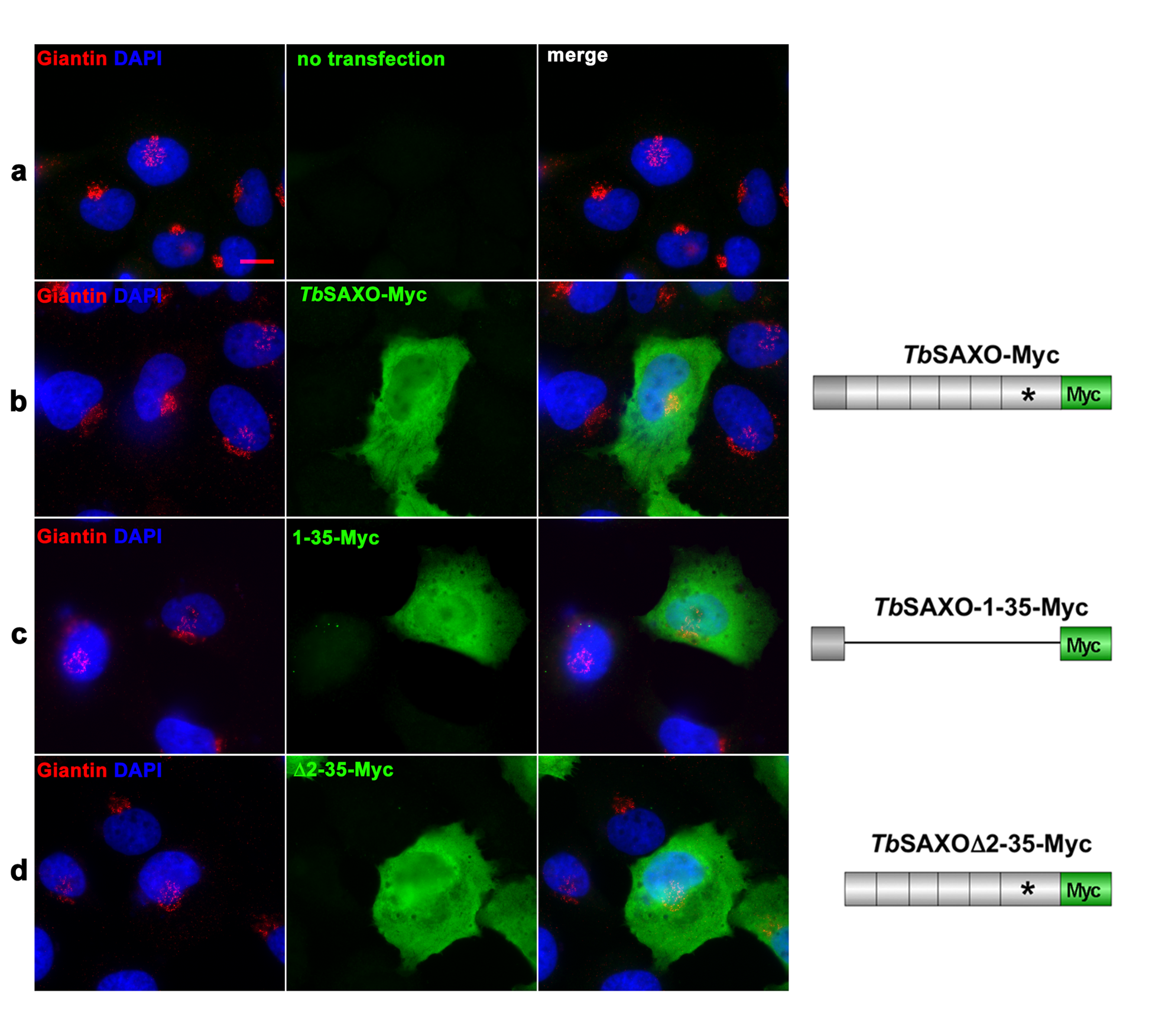

Supplement: Figure S6 — The N-terminus domain of Tb SAXO is not involved in Golgi targeting. Immuno-fluorescence on U-2 OS cells not transfected (a) or expressing (in green, labelled with anti-Myc) TbSAXO-Myc (b), the N-terminus (1–35)-Myc (c) or the deletion construct Δ2-35-Myc (d) co-stained with the Golgi marker anti-Giantin (in red) [108]. Nuclear DNA is labeled with DAPI. Bar, 8 µm. (TIF) [file pone.0031344.s006.tif]
